# High expression of eIF4E is associated with tumor macrophage infiltration and leads to poor prognosis in breast cancer

**DOI:** 10.1186/s12885-021-09010-0

**Published:** 2021-12-07

**Authors:** Fan Li, Huizhi Sun, Yue Li, Xiaoyu Bai, Xueyi Dong, Nan Zhao, Jie Meng, Baocun Sun, Danfang Zhang

**Affiliations:** 1 Department of Pathology, Tianjin Medical University, Tianjin, 300070 People’s Republic of China; 2 National Clinical Research Center for Cancer, Key Laboratory of Cancer Prevention and Therapy, Tianjin Medical University Cancer Institute and Hospital, Tianjin’s Clinical Research Center for Cancer, Tianjin, 300060 People’s Republic of China; 3 Department of Pathology, General Hospital of Tianjin Medical University, Tianjin, 300070 People’s Republic of China

**Keywords:** Eukaryotic translation initiation factor 4E (eIF4E), BRCA, Prognosis, Immune infiltration, Cytokines

## Abstract

**Background:**

The expression and activation of eukaryotic translation initiation factor 4E (eIF4E) is associated with cell transformation and tumor initiation, but the functional role and the mechanism whereby it drives immune cell infiltration in breast cancer (BRCA) remain uncertain.

**Methods:**

Oncomine, Timer and UALCAN were used to analyze the expression of eIF4E in various cancers. PrognoScan, Kaplan–Meier plotter, and GEPIA were utilized to analyze the prognostic value of eIF4E in select cancers. In vitro cell experiments were used to verify the role of eIF4E in promoting the progression of BRCA. ImmuCellAI and TIMER database were used to explore the relationship between eIF4E and tumor infiltrating immune cells. The expression of a macrophage marker (CD68^+^) and an M2-type marker (CD163^+^) was evaluated using immunohistochemistry in 50 invasive BRCA samples on tissue microarrays. The Human Protein Atlas (HPA) database was used to show the expression of eIF4E and related immune markers. LinkedOmics and NetworkAnalyst were used to build the signaling network.

**Results:**

Through multiple dataset mining, we found that the expression of eIF4E in BRCA was higher than that in normal tissues, and patients with increased eIF4E expression had poorer survival and a higher cumulative recurrence rate in BRCA. At the cellular level, BRCA cell migration and invasion were significantly inhibited after eIF4E expression was inhibited by siRNA. Immune infiltration analysis showed that the eIF4E expression level was significantly associated with the tumor purity and immune infiltration levels of different immune cells in BRCA. The results from immunohistochemical (IHC) staining further proved that the expression of CD68^+^ and CD163^+^ were significantly increased and correlated with poor prognosis in BRCA patients (*P* < 0.05). Finally, interaction network and functional enrichment analysis revealed that eIF4E was mainly involved in tumor-related pathways, including the cell adhesion molecule pathway and the JAK-STAT signaling pathway.

**Conclusions:**

Our study has demonstrated that eIF4E expression has prognostic value for BRCA patients. eIF4E may act as an essential regulator of tumor macrophage infiltration and may participate in macrophage M2 polarization.

**Supplementary Information:**

The online version contains supplementary material available at 10.1186/s12885-021-09010-0.

## Background

BRCA is the most common malignancy in women worldwide and is curable in 70–80% of patients with early, non-metastatic disease. Advanced BRCA with distal organ metastases is considered incurable with currently available treatments [1]. In 2019, approximately 316,700 new BRCA cases were diagnosed in women in the United States, an annual increase of nearly 0.3%. Data from China show that the incidence of BRCA is also increasing every year (272,400 cases in 2015 and 367,900 cases in 2018) [2, 3]. BRCA is considered to be comprised of at least four different clinically relevant molecular subtypes: luminal A, luminal B, Her2-enriched, and basal-like subtypes [4, 5].

eIF4E is one of the essential constituents of the protein translation initiation factor complex (eukaryotic translation initiation factor 4, eIF4F) in the eukaryotic protein translation initiation machinery. eIF4E recognizes and binds mRNA caps containing 7-methylguanosine in the early stages of protein synthesis, and promotes ribosome binding by inducing the release of the secondary structure of mRNA. As a proto-oncogene, the expression and activation of eIF4E are associated with cell transformation and tumor initiation. Translation of mRNA transcripts can be selectively regulated by specific RNA binding proteins and microRNAs and also by regulating the 5′-cap binding activity of eIF4E [6, 7]. Previous studies have shown that patients with high expression of eIF4E are more likely to relapse and have higher mortality than those with minimal expression of eIF4E in triple-negative BRCA (TNBC) [8].

In this study, we used a variety of databases to explore the expression of eIF4E and its impact on the prognosis in BRCA. In vitro cell experiments were used to verify the role of eIF4E in promoting the progression of BRCA. We further explored the relationship between eIF4E and tumor infiltrating immune cells using ImmuCellAI and the TIMER database. The expression of macrophage markers was evaluated using immunohistochemistry in 50 invasive BRCA samples on tissue microarrays. Our results provide new insights into the functional role of eIF4E in BRCA.

## Methods

### Clinical samples

We collected 50 samples from BRCA patients, including detailed pathological and clinical information. All patients underwent surgery in Tianjin General Hospital between 1997 and 2004. The median age of the patients was 48 years (36 to 69 years). All patients had invasive breast cancer, and axillary node metastases were present in 15 patients. The diameter of the primary tumor in 8 patients was < 2 cm, and > 5 cm in 2 patients. The follow-up period started at the time of the surgery and ended in December 2008.

### Cell culture and reagents

The human breast cancer cell line MDA-MB-231 was cultured in DMEM supplemented with 10% fetal bovine serum (Invitrogen, CA, USA) and 1% penicillin-streptomycin in an incubator at 37 °C with 5% CO2. All secondary antibodies were purchased from Zhongshan Golden Bridge Biotechnology Co., Ltd.

### Stable transfection using lentiviral infection

The PLKO.1-puro vector was used to clone the sh-RNA targeting eIF4E. The sequence of sh-eIF4E was 5′-CCGGCCAAAGATAGTGATTGGTTATCTCGAGATAACCAATCACTATCTTTGGTTTTTG-3′. The plasmids were transfected into HEK293T cells, and the supernatant containing the virus was collected at 48 h. The virus was then concentrated and transfected into breast cancer cells with polybrene. The transfected cells were selected by puromycin for at least 1 week. To obtain stable control cell lines, breast cancer cells were transfected with empty lentiviral vectors.

### Western blotting analysis

Protein was extracted using SDS lysis buffer and transferred to PVDF membranes. After the membranes were blocked with 5% skim milk for 1 h, they were incubated with primary antibodies overnight at 4 °C, which was followed by incubation with secondary antibodies for 2 h. Bands were visualized using a C-Digit Blot Scanner (Gene Company) and analyzed with ImageJ software. GAPDH (sc-47,724, 1:1000; Santa Cruz) was used as a protein loading control. Primary antibody against eIF4E (sc-9976, 1:500) was purchased from Santa Cruz.

### Wound-healing assay

Cells were seeded in 24-well plates. When cells reached confluency, a wound was created using a 100-μL sterile pipette tip and photographed (0 h). The rate of gap closure was measured at various time points. Each experiment was performed three times.

### Cell invasion assay and cell migration assays

For migration assays, breast cancer cells (1 × 10^5^) were suspended in serum-free medium and added to the upper chamber of the Transwell plate. DMEM with 10% FBS was added to the bottom chamber in 24-well plates. After the cells were incubated for 24 h, they were fixed with methanol and stained with crystal violet for 20 min. Invasion assays were performed the same say as the migration assays except that the Transwell chambers were coated with Matrigel before the cells were seeded in the upper chamber. These cells were counted using an inverted light microscope (Nikon). Each experiment was performed three times.

### Immunohistochemistry

The tissues were deparaffinized in xylene and rehydrated in graded alcohols. First, 3% H_2_O_2_ was used to block endogenous peroxidase, followed by antigen retrieval. Tissue sections were blocked in 10% goat serum (Zhongshan Chemical Co., Beijing, China) and incubated consecutively with primary antibodies and a secondary antibody. Mouse anti-human CD68^+^ monoclonal antibody (zm-0060) and mouse anti-human CD163^+^ monoclonal antibody (zm-0428) were obtained from Beijing Zhongshan Jinqiao Biotechnology Company. Known positive tissue sections were used as a positive control, and PBS was used instead of primary antibody as a negative control. On the immunohistochemistry sections, CD68^+^ and CD163^+^ were expressed in the cytoplasm of macrophages, and the positive macrophages were identified by yellow brown or brown granules. First, the whole section was observed under a low-power (100×) optical microscope. Five non-overlapping high-power fields (400 times) in the stroma were selected to count the positive cells. The average number of positive cells in the five fields was recorded as the final result for the sample. Based on the staining results, the best cut-off value was selected as the dividing point of high and low expression. The number of CD68^+^ macrophages in tumor nests(TN)less than 14.4 was defined as low expression and more than 14.4 as high expression; the number of CD68^+^ macrophages in tumor stroma (TS) less than 18.6 was defined as low expression and more than 18.6 as high expression; the number of CD163^+^ macrophages in TN less than 19.58 was defined as low expression, the number of CD163^+^ macrophages in TS less than 27.6 was defined as low expression, and the number of CD163^+^ macrophages more than 27.6 was defined as high expression.

### Oncomine database analysis

The Oncomine database compiled 86,733 samples and 715 gene expression datasets into a single comprehensive database designed to facilitate data mining efforts [9]. We therefore used this database to assess the association between eIF4E expression and prognostic outcome in various cancer types (https://www.oncomine.org/resource/login.html).

### PrognoScan database analysis

The PrognoScan database is designed to facilitate meta-analyses of the prognostic value of the gene by comparing the relationship between gene expression and relevant outcomes, including overall survival (OS), in a wide range of published cancer microarray datasets [10]. We therefore used this database to assess the relationship between eIF4E expression and patient outcome (http://www.abren.net/PrognoScan/).

### Kaplan–Meier plotter analysis

The Kaplan–Meier plotter offers a means of readily exploring the impact of a wide array of genes on patient survival in 21 different types of cancer, with large sample sizes for the breast (*n* = 6234), ovarian (*n* = 2190), lung (*n* = 3452) and gastric (*n* = 1440) cancer cohorts [11]. We therefore used this database to explore the association between eIF4E expression and outcome in patients with gastric, breast, ovarian and lung cancers (http://kmplot.com/analysis/).

### TIMER database analysis

TIMER (https://cistrome.shinyapps.io/timer/) is a database designed for the analysis of immune cell infiltrates in multiple cancers. This database employs pathological examination-validated statistical methodology to estimate infiltration by neutrophils, macrophages, dendritic cells, B cells and CD4^+^/CD8^+^ T cells into tumors [12]. We initially employed this database to assess differences in eIF4E expression levels in specific tumor types using the TIMER database, and then explored the association between eIF4E expression and the degree of infiltration by specific immune cell subsets. Then, Kaplan–Meier curve analysis and a multifactor Cox proportional hazard model were carried out to explore the effect of immune cell infiltration on the survival rate of breast cancer patients. Finally, the relationship between the expression of eIF4E and the expression of specific infiltrating immune cell subsets was evaluated.

### GEPIA database analysis

GEPIA (http://gepia.cancer-pku.cn/index.html) is an online database that can be used for standardized TCGA and GTEx dataset analysis of tumor samples and control samples [13]. The GEPIA database was used to evaluate the relationship between the expression of eIF4E and the prognosis of patients, as well as the subgroup analysis based on clinical pathological features.

### ImmuCellAI database analysis

The ImmuCellAI tool can accurately predict the abundance of 24 types of immune cells in the sample, including 18 T cell subtypes [14], based on the expression data of RNA-Seq or microarray. We used the Gene Expression Omnibus (GEO; http://www.ncbi.nlm.nih.gov/geo/) dataset GSE109169 to analyze the difference in gene expression between breast cancer tissues and normal tissues adjacent to the cancer and estimated the extent of immune cell infiltration by using the ImmuCellAI data bank (http://bioinfo.life.hust.edu.cn/web/ImmuCellAI/).

### LinkedOmics database analysis

The LinkedOmics database (http://www.linkedomics.org/login.php) is a web-based platform for analyzing 32 TCGA cancer-associated multi-dimensional datasets [15]. EIF4E coexpression was analyzed statistically using Pearson’s correlation coefficient and is presented in volcano plots, heat maps, or scatter plots. The functional module of LinkedOmics analyzed gene ontological biological process (GO_BP), KEGG pathway, kinase target enrichment, miRNA target enrichment and transcription factor target enrichment by gene set enrichment analysis (GSEA).

### NetworkAnalyst database analysis

To interpret gene expression networks, the NetworkAnalyst 3.0 tool was used (https://www.networkanalyst.ca/) [16]. This tool integrates cell-type or tissue-specific protein-protein interaction (PPI) networks, gene regulatory networks, and gene coexpression networks.

### Enrichment analysis of GO and KEGG pathways

The database (DAVID v.6.8) and the database (DAVID.ncifcrf.gov/) were used to identify the enrichment analysis [17]. The GO biological process analysis and the KEGG pathway enrichment analysis were carried out on the key genes of coexpression and immune marker gene cross, and visualized by Cytoscape v3.7.2 software [18] and R4.0.2 language. The *P* value adjusted by FDR was statistically significant.

### Human protein atlas database

The expression of eIF4E and related immune markers in BRCA was verified using the HPA database (https://www.proteinatlas.org/). Protein expression in 44 major human tissues and some tumor tissues was determined by the IHC method [19].

### Statistical analysis

Statistical analyses were performed with SPSS 22.0 (SPSS Inc., Chicago, IL, USA). Values are expressed as the mean ± SD (standard deviation). The chi-squared test was used to compare categorical variables. The PrognoScan, Kaplan–Meier plotter, TIMER and GEPIA databases were used to generate survival plots in the respective analyses, with data including either HR and *P* values or P values derived from a log-rank test. Data from the Oncomine database are presented with information regarding ranking, fold-change and P values. Pearson or Spearman’s correlation analyses were used to gauge the degree of correlation between particular variables, with the following r values being used to judge the strength of correlation: 0.00–0.19 ‘very weak’, 0.20–0.39 ‘weak’, 0.40–0.59 ‘moderate’, 0.60–0.79 ‘strong’, and 0.80–1.0 ‘very strong’. *P* < 0.05 was the significance threshold.

## Results

### Expression of eIF4E in various tumors and normal tissues

**Fig. 1:**
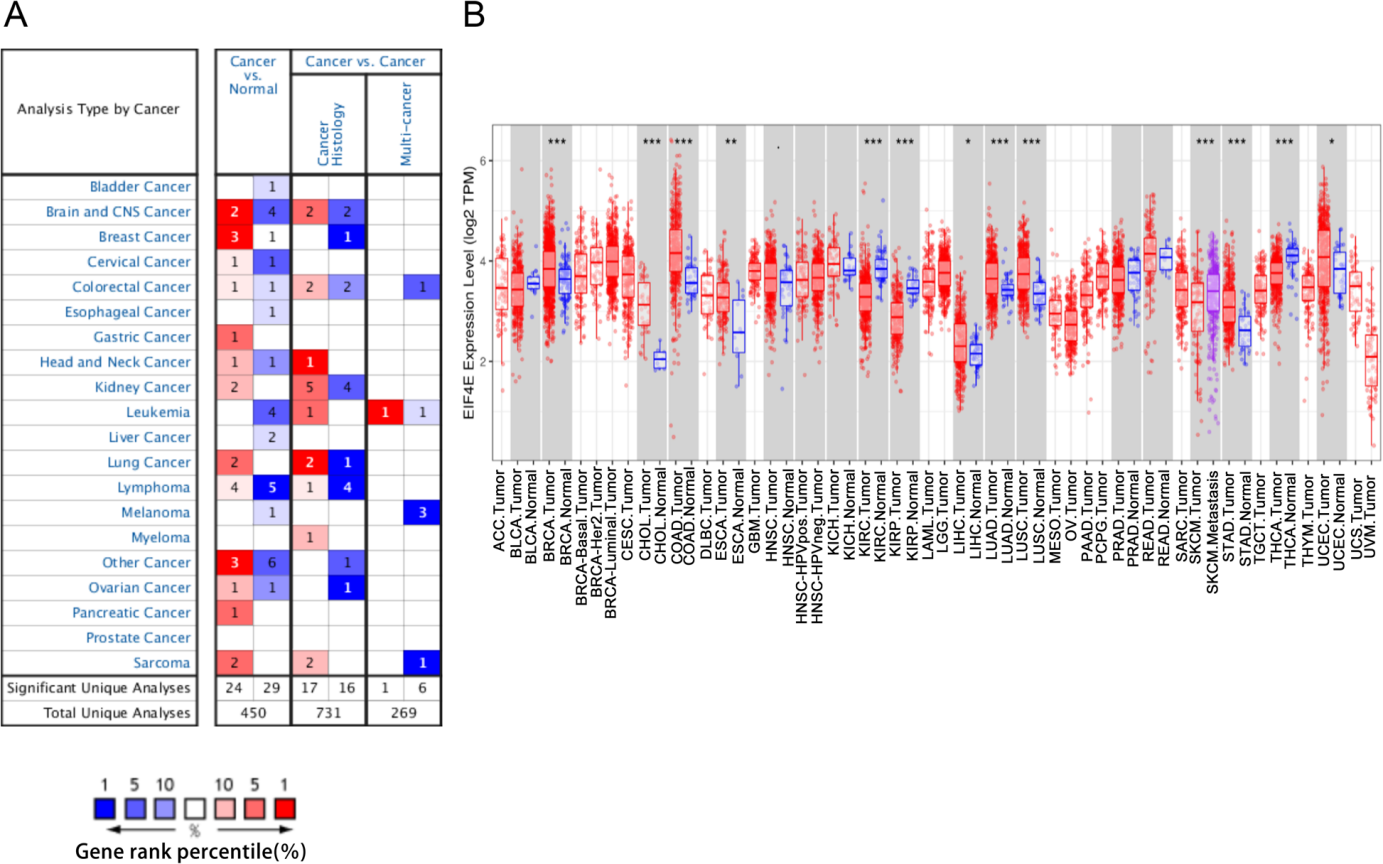
The expression level of eIF4E in different types of tumor tissues and normal tissues (**A**) The expression level of eIF4E in different types of tumor tissues and normal tissues in the Oncomine database. (*P* value is .001, fold change is 1.5, and gene ranking of all). **B** The expression level of eIF4E in different types of tumor tissues and normal tissues in TIMER database (*, *p* < 0.05; **, *p* < 0.01; ***, *p* < 0.001)

We first analyzed the expression of eIF4E in a variety of tumors and normal tissues using the Oncomine database and found that the expression of eIF4E in brain cancer, BRCA, cervical cancer, colorectal cancer, gastric cancer, head and neck tumor, kidney cancer, lung cancer, lymphoma, ovarian cancer, pancreatic cancer, sarcoma and other tumors was higher than that in normal tissues (*P* < 0.001) (Fig. 1A). The mRNA-seq data from TCGA were analyzed using TIMER to verify these findings (Fig. 1B). Data from TCGA showing the differential expression of eIF4E between the tumor and adjacent normal tissues are shown in Fig. 1B. Compared with adjacent normal tissues, eIF4E expression was significantly upregulated in BRCA, cholangiocarcinoma (CHOL), colon adenocarcinoma (COAD), esophageal carcinoma (ESCA), head and neck squamous cell carcinoma (HNSC), liver hepatocellular carcinoma (LIHC), lung adenocarcinoma (LUAD), lung squamous cell carcinoma (LUSC), stomach adenocarcinoma (STAD), and uterine corpus endometrial carcinoma (UCEC). The expression of eIF4E in thyroid carcinoma (THCA), kidney renal papillary cell carcinoma (KIRP), and kidney renal clear cell carcinoma (KIRC) was significantly lower than that in the normal control samples. (*P* < 0.05).

**Fig. 2:**
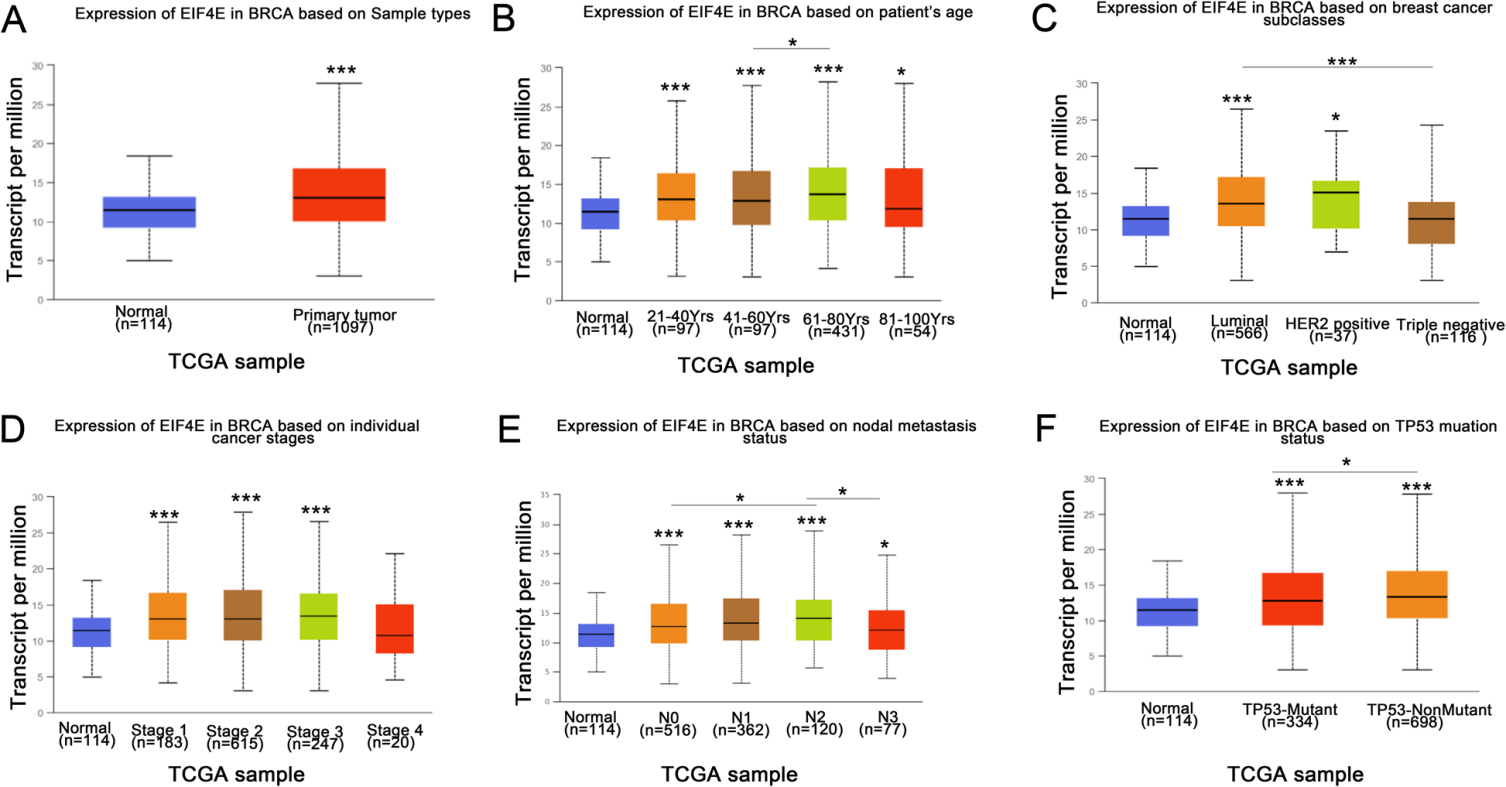
eIF4E transcription in subgroups of patients with BRCA, stratified based on gender, age and other criteria (UALCAN). Box-whisker plots showing the expression of eIF4E in sub groups of BRCA samples. **A** Boxplot showing relative expression of eIF4E in normal and BRCA samples. **B** Boxplot showing relative expression of eIF4E in normal individuals of any age or in BRCA patients aged 21–40, 41–60, 61–80, or 81–100 yr. **C** Boxplot showing relative expression of eIF4E in BRCA based on breast cancer subclasses. **D** Boxplot showing relative expression of eIF4E in normal individuals or in BRCA patients in stages 1, 2, 3 or 4. **E** Boxplot showing relative expression of eIF4E in BRCA based on nodal metastasis status. **F** Boxplot showing relative expression of eIF4E in BRCA based on TP53 mutation status. The central mark is the median; the edges of the box are the 25th and 75th percentiles. The t-test was used to estimate the significance of difference in gene expression levels between groups. *, *p* < 0.05; **, *p* < 0.01; ***, *p* < 0.001

Further subgroup analysis of multiple clinicopathological features of TCGA-Breast invasive carcinoma samples in the UALCAN database consistently showed an increase in the transcript level of eIF4E. According to the analysis of sample type, age, subtype of BRCA, disease stage, lymph node metastasis and TP53 mutation, the expression of eIF4E in BRCA patients was significantly higher than that in normal controls, and the expression of eIF4E in patients aged 61 to 80 was significantly higher than that in patients aged 41 to 60 (*P* = 0.037399). In all subtypes of BRCA, the expression of eIF4E was significantly higher than that in normal subjects, the expression in the luminal type was significantly higher than that in TNBC (*P* < 0.01), and the expression levels in tumor stages 1, 2 and 3 were significantly higher than those in the normal group. Lymph node metastasis showed that the expression level of N2 was the highest and significantly different from that of N0 (*P* = 0.0127978) and N3 (*P* = 0.0169045). TP53 mutation analysis showed that the expression level of eIF4E in the TP53 non-mutated group was higher than that in the mutant group (*P* = 0.024296) (Fig. 2). Therefore, according to the differences in BRCA subtypes, tumor stages and lymph node metastasis, the expression of eIF4E can be used as a prognostic biomarker in BRCA.

### Relationship between eIF4E expression and prognosis of patients with various tumors

**Fig. 3:**
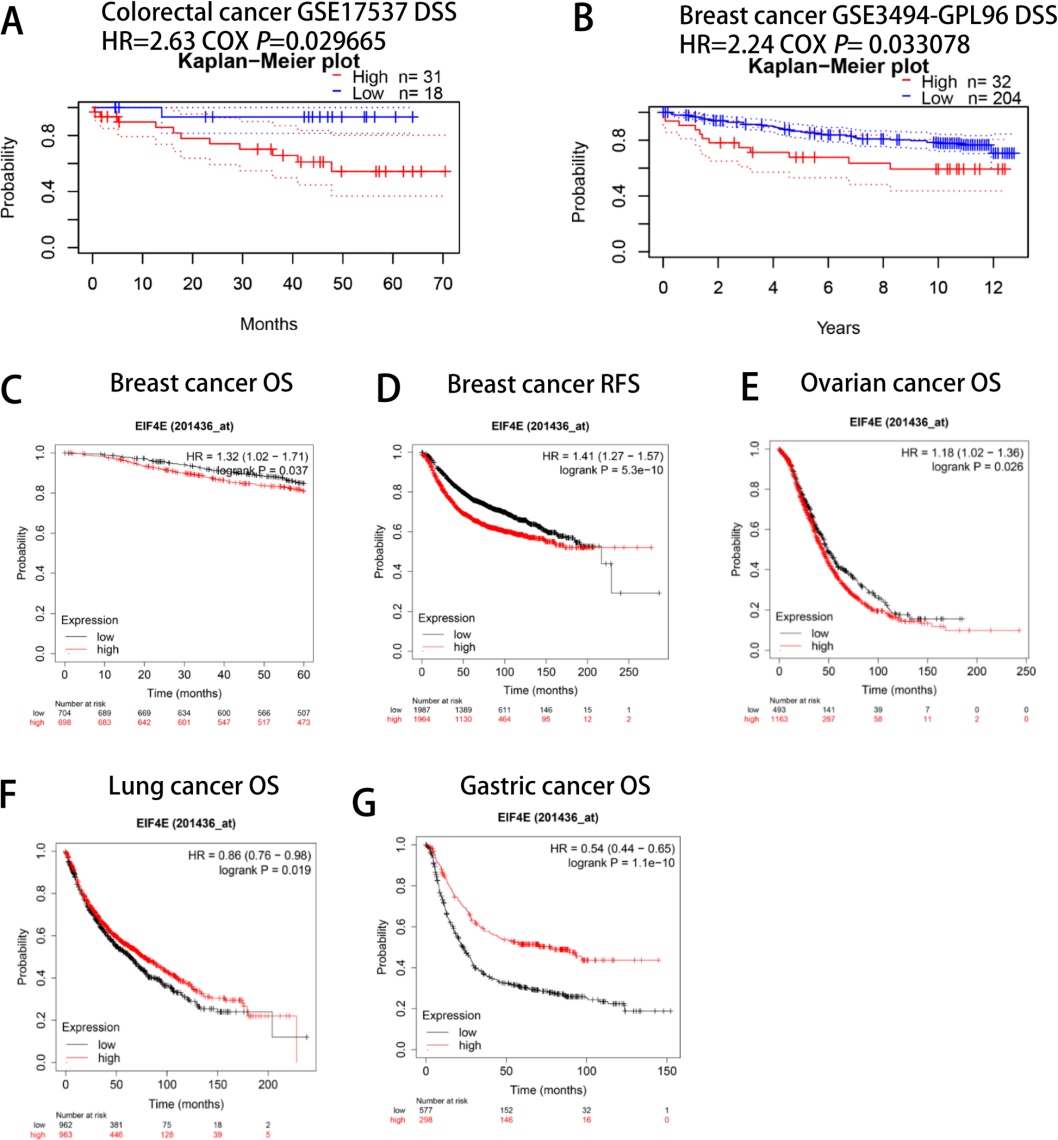
Correlation between eIF4E and prognosis of various types of cancer Correlation between eIF4E and prognosis of various types of cancer in the PrognoScan (**A–B**) Correlation between eIF4E and prognosis of various types of cancer in the Kaplan-Meier plotter database (**C–G**). OS, overall survival; DSS,disease free survival; RFS, recurrence-free survival

Next, we used the PrognoScan database to explore the relationship between the expression of eIF4E and the prognosis of tumor patients. We found that breast and colorectal cancers were significantly associated with the expression of eIF4E (Fig. 3A, B) (DSS: disease-specific survival; RFS: relapse-free survival). In addition, we used the Kaplan–Meier database to evaluate the relationship between the expression of eIF4E in a range of tumor types and prognosis. The results showed that the increased expression of eIF4E was significantly correlated with the poor prognosis in BRCA (OS HR = 1.32, 95% CI =1.02–1.71, *P* = 0.037; HR =1.41, 95% CI =1.27–1.857, *P* = 5.3e-10). The increased expression of eIF4E was also significantly associated with poor prognosis in ovarian cancer (OS HR = 1.1810, 95% CI = 1.02–1.36, *P* = 0.026). However, in lung and gastric cancers, decreased expression of eIF4E was significantly associated with poor prognosis (lung cancer OS HR =0.86, 95% CI =0.76–0.98, *P* = 0.019; gastric cancer OS HR =0.54, 95% CI =0.44–0.65, *P* = 1.1e-10) (Fig. 3C-G). We further used the GEPIA database to evaluate the relationship between the expression of eIF4E and the prognosis of patients and analyzed 33 tumor types. It was found that the prognosis of patients with high expression of eIF4E was poor in BRCA, brain low-grade glioma, lung adenocarcinoma, cervical squamous cell carcinoma and adenocarcinoma, hepatocellular carcinoma and lung squamous cell carcinoma, while the low expression of eIF4E in renal clear cell carcinoma, hepatic clear cell carcinoma and colorectal cancer had poor prognosis (Fig. S1A-I). These results clearly showed that in many tumor types, the expression of eIF4E was significantly correlated with poor prognosis, and the high expression of eIF4E in various databases was significantly correlated with poor prognosis of BRCA patients.

### Knocking down eIF4E inhibited cell migration and invasion in MDA-MB-231 cells

**Fig. 4:**
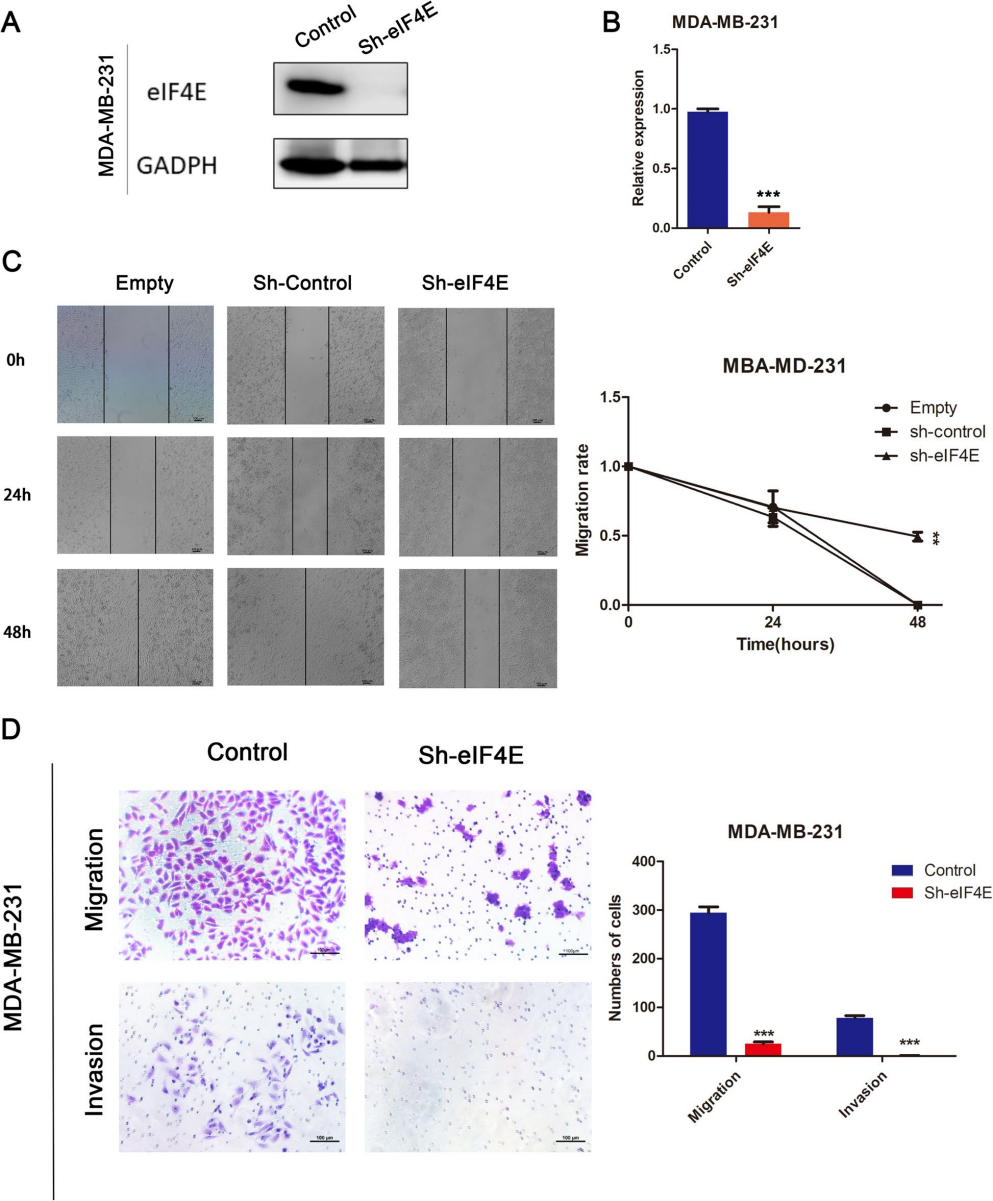
Knocking down eIF4E inhibited cell migration and invasion in MDA-MB-231. **A** Western blotting and quantitative analysis (**B**) of MDA-MB-231-SheIF4E, MDA-MB-231-control cells (Student’s t-test, *p* < 0.05). **C** Wound healing assays of MDA-MB-231-sheIF4E, MDA-MB-231-control and empty cells. **D** Migration and invasion assay of MDA-MB-231-sheIF4E, MDA-MB-231-control and empty cells. *, *p* < 0.05; **, *p* < 0.01; ***, *p* < 0.001

To comprehend the function of eIF4E in BRCA cells, we modulated the levels of eIF4E expression by shRNA-based techniques (in TNBC MDA-MB-231 cells). Using Western blotting (Student’s t-test, *p* < 0.05), we confirmed that the expression of eIF4E was decreased in MDA-MB-231 cells treated with siRNA (Fig. 4A, B). Wound healing and Matrigel-coated (for invasion) or Matrigel-uncoated (for migration) Transwell analysis showed that eIF4E knockdown effectively inhibited the invasion and migration of BRCA cells (Fig. 4C, D). Wound healing assays and quantitative analysis demonstrated that the downregulation of eIF4E inhibited the migration of MDA-MB-231 cells. Moreover, in the migration assay presented in Fig. 4D, a decrease in cell migration was observed in the Sh-eIF4E MDA-MB-231 cell line compared with the negative vector control. Similarly, similar results were observed in the Matrigel invasion assay. These results reveal the role of eIF4E in promoting the progression of BRCA in vitro.

### Relationship between eIF4E expression and infiltration of immune cells in BRCA

**Fig. 5:**
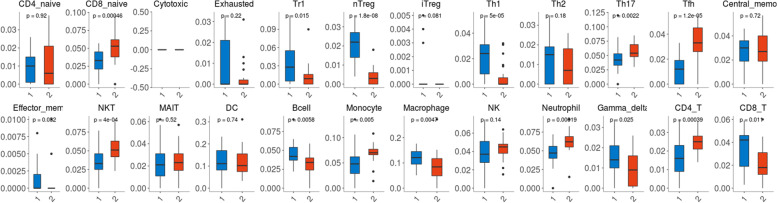
Immune cell abundance analysis between the breast cancer tumor tissues and adjacent normal tissues in GSE109169 to estimate the abundance of immune cell infiltration. *, *p* < 0.05; **, *p* < 0.01; ***, *p* < 0.001.(blue box: breast cancer group; red box: normal group)

The gene expression dataset GSE109169, related to BRCA, was searched from the comprehensive gene expression database (GEO) to analyze the difference in gene expression between BRCA and adjacent normal tissues (Supplementary Table 1). The abundance of immune cell infiltration was calculated by the ImmuCellAI database. It was found that, among 18 subtypes of T cells and 6 other types of immune cells, the infiltration levels of macrophages, nTreg cells, Th1 cells, B cells, CD8^+^ T cells and γδT cells in BRCA tissues were significantly higher than those in adjacent normal tissues. The infiltration levels of Th17 cells, Tfh cells, NKT cells, monocytes, neutrophils and CD4^+^ T cells in tumor tissues were lower than those in normal tissues (Fig. 5). This showed that there are significant differences in immune cell infiltration between BRCA and adjacent normal tissues, and different levels of immune cell infiltration have potential effects on the tumor initiation, progression and survival of BRCA patients.

**Fig. 6:**
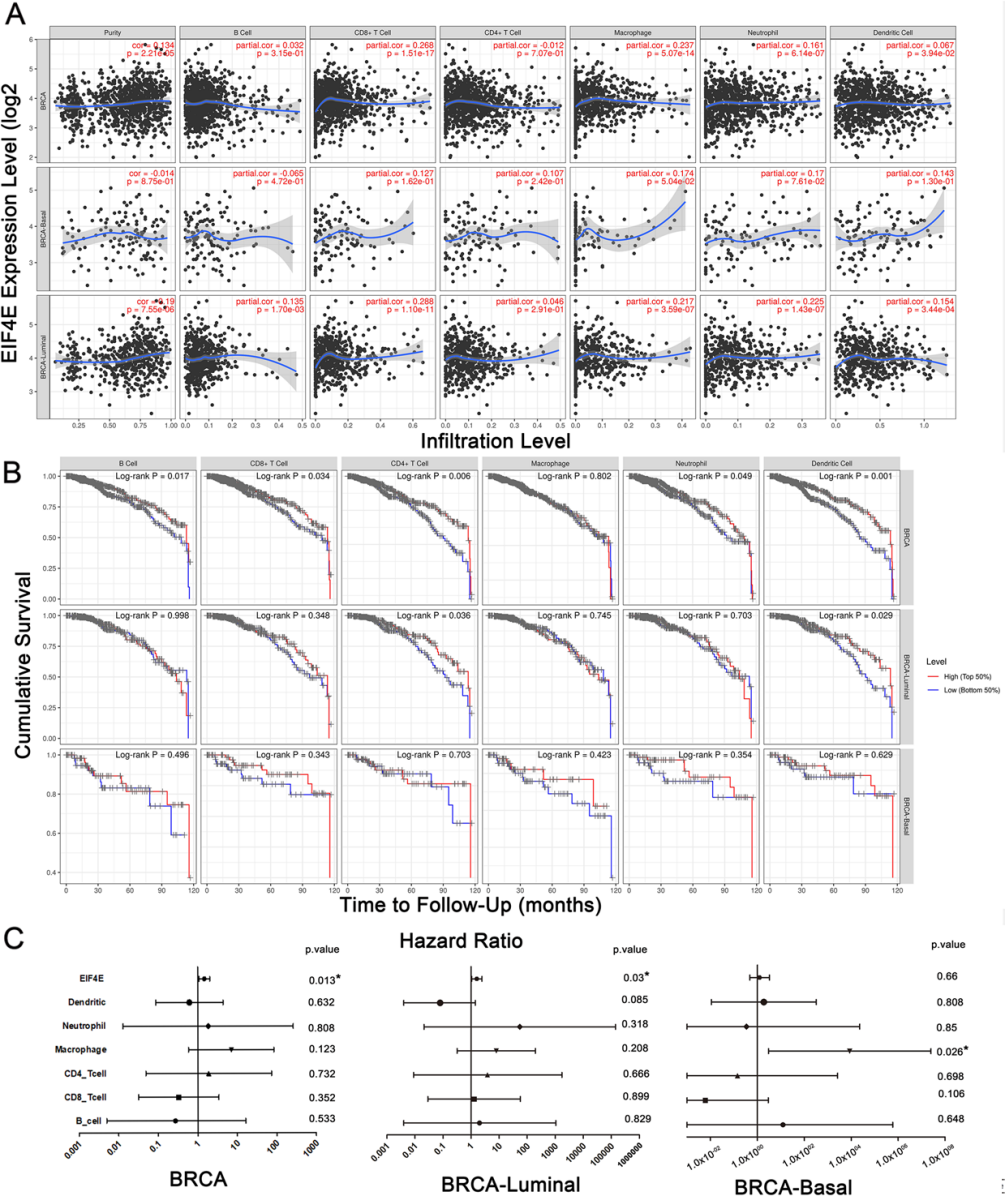
eIF4E expression is correlated with the level of immune infiltration in BRCA. **A** eIF4E expression is correlated with the level of immune infiltration in BRCA, BRCA-Luminal and BRCA-Basal. **B** Kaplan-Meier plots of immune infiltration in BRCA, BRCA-Luminal and BRCA-Basal. **C** Multivariable hazards models were used to evaluate the impacts of eIF4E expression on survival in the presence of infiltrating levels of multiple immune cells. *, *p* < 0.05; **, *p* < 0.01; ***, *p* < 0.001

Since we found that the expression of eIF4E was related to the poor prognosis of patients with BRCA, we further drew a Kaplan–Meier map using the TIMER database to explore the relationship between immune cell infiltration and the expression of eIF4E and investigated its potential mechanism in BRCA. In BRCA in general, eIF4E expression was significantly correlated with tumor purity (*r* = 0.134, *P* = 2.2e-05), CD8^+^ T cells (*r* = 0.268, *P* = 1.5e-17), macrophages (*r* = 0.237, *P* = 5.1e-14), neutrophils (*r* = 0.161, *P* = 6.1e-07) and dendritic cells (*r* = 0.067, *P* = 3.9e-02). In BRCA-Basal, the expression of eIF4E was significantly correlated with macrophage infiltration (*r* = 0.174, *P* = 5.0e-02); In BRCA-Luminal, the expression of eIF4E was significantly correlated with B cells (*r* = 0.135, *P* = 1.7e-03), CD8^+^ T cells (*r* = 0.288, *P* = 1.1e-11), macrophages (*r* = 0.217, *P* = 3.6e-07), neutrophils (*r* = 0.225, *P* = 1.4e-07) and dendritic cells (*r* = 0.154 *P* = 3.4e-04) (Fig. 6A). Macrophage infiltration was consistent with the expression of eIF4E in basal and luminal subtypes.

To further study the relationship between immune cell infiltration and eIF4E expression in BRCA, we used the TIMER database to generate a Kaplan–Meier map. We found that the infiltration of CD8^+^ T cells (*P* = 0.006), CD4^+^ T cells (*P* = 0.006), neutrophils (*P* = 0.007) and dendritic cells (*P* = 0.004) was significantly correlated with the prognosis of BRCA in general (Fig. 6B). As shown in Fig. 6C, proportional risk model analysis showed that eIF4E acted as an independent prognostic factor in the presence of a variety of infiltrating immune cells in BRCA (HR = 1.482, *P* = 0.013) and BRCA-Luminal (HR = 1.597, *P* = 0.03); in BRCA-Basal, macrophage infiltration acted as an independent prognostic factor (HR = 8643.368, *P* = 0.026). This suggested that eIF4E played an important role in regulating immune cell infiltration in BRCA.

### Evaluation of the correlation between eIF4E and the expression of immune markers

**Fig. 7:**
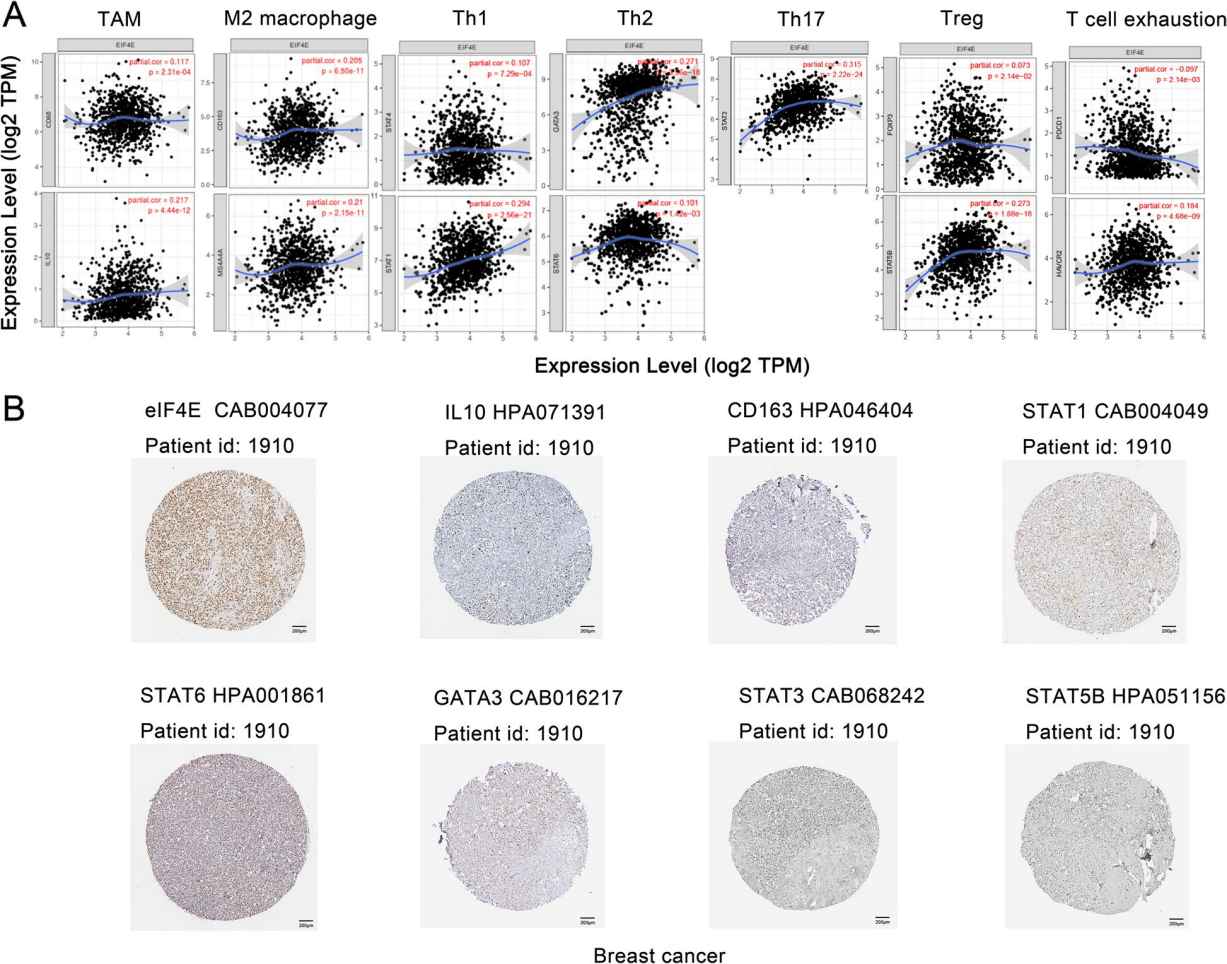
Correlation analysis between eIF4E and immune marker expression. **A** Scatterplots of correlations between eIF4E expression and gene markers of TAMs, M2 macrophages and Th1 and Th2 and Th17 and Treg and T cell exhaustion in BRCA. **B** Immunohistochemical map of HPA database showing significant expression levels of eIF4E related immune cell markers(Scale bar,200 μm)

Next, we used TIMER databases to further explore the relationship between the expression of eIF4E and the level of immune cell infiltration in BRCA. We evaluated the correlation between eIF4E expression and specific cell subsets, including CD8^+^ T cells, B cells, monocytes, TAMs, M1 and M2 macrophages, neutrophils, NK cells, dendritic cells (DCs), Th1 cells, Th2 cells, Tfh cells, Th17 cells, regulatory T cells (Tregs) and exhausted T cells. We adjusted these results according to the purity of the tumor. Markers for CD8^+^ T cells (CD8B), B cells (CD19, CD79A), monocytes (CD86), TAMs (CCL2, CD68, IL10), M1 macrophages (COX2), M2 macrophages (CD163, VSIG4, MS4A4A), neutrophils (CD11b), DCs (HLA-DPB1, HLA-DRA, HLA-DPA1, BCDA-4), Th1 cells (STAT4, STAT1), Th2 cells (GATA3, STAT6), Tfh cells (BCL6, IL21), Th17 cells (STAT3), Tregs (FOXP3, CCR8, STAT5B), and exhausted T cells (PD-1, LAG3, TIM-3) were significantly correlated with eIF4E expression (Table 1). The expression of eIF4E in BRCA was positively correlated with the expression of markers for monocytes, TAMs, M1 macrophages, M2 macrophages, neutrophils, Th1 cells, Th2 cells, Tfh cells, Th17 cells and Tregs and negatively correlated with those for CD8^+^ T cells, B cells, dendritic cells and exhausted T cells. Figure 7A shows the scatter diagram of TAM, M2 macrophage, Th1, Th2, Th17, Treg and exhausted T cell markers.

**Table 1:** Correlation analysis between eIF4E and relate genes and markers of immune cells in TIMER

Description	Gene makers	Breast cancer			
		None		Purity	
		Cor	***P***	partial.cor	partial.***p***
CD8 + T cell	CD8A	− 0.022	4.75 × 10–1	0.04686004	0.13985143
	CD8B	− 0.148	8.28 × 10–7^***^	− 0.10188943	0.00129684^*^
T cell (general)	CD3D	−0.125	3.09 × 10–5^***^	−0.06614794	0.03705509^*^
	CD3E	−0.094	1.82 × 10–3^*^	−0.02731704	0.38961155
	CD2	−0.043	1.5 × 10–1	0.02692737	0.3964128
B cell	CD19	−0.146	1.08 × 10–6^***^	−0.09601416	0.00244306^*^
	CD79A	−0.136	5.99 × 10–6^***^	−0.0761307	0.01636396^*^
Monocyte	CD86	0.096	1.47 × 10–3^*^	0.15738691	6.1348E-07^***^
	CD115(CSF1R)	0.001	9.73 × 10–1	0.0607296	0.05561681
TAM	CCL2	0.002	9.39 × 10–1	0.06986978	0.02761177^*^
	CD68	0.065	3.01 × 10–2	0.11656366	0.00023057^**^
	IL10	0.153	3.38 × 10–7^***^	0.21721067	4.4418E-12^***^
M1 Macrophage	INOS (NOS2)	−0.024	4.34 × 10–1	−0.0130669	0.68072849
	IRF5	0.016	5.91 × 10–1	0.03883945	0.22116287
	COX2(PTGS2)	0.043	1.56 × 10–1	0.12822816	5.0261E-05^***^
M2 Macrophage	CD163	0.152	4.1 × 10–7^***^	0.20522705	6.4959E-11^***^
	VSIG4	0.077	1.04 × 10–2	0.12544706	7.3161E-05^***^
	MS4A4A	0.138	4.39 × 10–6^***^	0.210251	2.1511E-11^***^
Neutrophils	CD66b(CEACAM8)	−0.018	5.52 × 10–1	−0.01102645	0.72843176
	CD11b(ITGAM)	0.041	1.76 × 10–1	0.08676308	0.00619708^*^
	CCR7	−0.055	6.61 × 10–2	0.0154633	0.62630164
Natural killer cell	KIR2DL1	−0.015	6.12 × 10–1	0.00357961	0.9102562
	KIR2DL3	−0.008	7.97 × 10–1	0.0146039	0.64560698
	KIR2DL4	−0.027	3.62 × 10–1	0.00685601	0.8290771
	KIR3DL1	−0.04	1.82 × 10–1	−0.00907876	0.77497103
	KIR3DL2	−0.051	9.37 × 10–2	−0.01393341	0.66083692
	KIR3DL3	0.004	8.82 × 10–1	0.01473616	0.64262004
	KIR2DS4	−0.028	3.57 × 10–1	0.01435843	0.65116604
Dendritic cell	HLA-DPB1	−0.167	2.46 × 10–8^***^	−0.11866431	0.000177^**^
	HLA-DQB1	−0.113	1.75 × 10–4^**^	−0.05129112	0.10606764
	HLA-DRA	0.011	7.1 × 10–1	0.08384234	0.0081765^*^
	HLA-DPA1	0.011	7.12 × 10–1	0.08372552	0.00826629^*^
	BCDA-1(CD1C)	−0.083	5.95 × 10–3^*^	−0.0070813	0.82355157
	BCDA-4(NRP1)	0.144	1.59 × 10–6^***^	0.20736387	4.0736E-11^***^
	CD11c(ITGAX)	−0.02	5.17 × 10–1	0.04743547	0.1350466
Th1	T-bet (TBX21)	−0.096	1.45 × 10–3^*^	−0.03946619	0.21379428
	STAT4	0.024	4.34 × 10–1	0.10698215	0.00072919^**^
	STAT1	0.275	1.62 × 10–20^***^	0.29435209	2.558E-21^***^
	IFN-γ (IFNG)	−0.033	2.75 × 10–1	0.0081502	0.79745787
	TNF-α (TNF)	−0.068	2.51 × 10–2	−0.03221895	0.31021222
Th2	GATA3	0.301	1.66 × 10–24^***^	0.27086187	3.5639E-18^***^
	STAT6	0.075	1.32 × 10–2	0.10105935	0.00142107^*^
	STAT5A	0.001	9.78 × 10–1	0.04334184	0.17212936
	IL13	−0.009	7.67 × 10–1	0.02091229	0.51017828
Tfh	BCL6	0.062	4.01 × 10–2	0.10660096	0.00076196^**^
	IL21	0.041	1.71 × 10–1	0.07605566	0.01647037^*^
Th17	STAT3	0.304	5.92 × 10–25^***^	0.31529031	2.2229E-24^***^
	IL17A	−0.007	8.23 × 10–1	0.01164907	0.71375374
Treg	FOXP3	0.006	8.53 × 10–1	0.07298812	0.02137316^*^
	CCR8	0.217	3.19 × 10–13^***^	0.27040878	4.0704E-18^***^
	STAT5B	0.254	1.35 × 10–17^***^	0.27303179	1.8794E-18^***^
	TGFβ (TGFB1)	−0.093	1.95 × 10–3^*^	−0.0489941	0.12267125
T cell exhaustion	PD-1(PDCD1)	−0.142	2.14 × 10–6^***^	−0.09726802	0.00214011^*^
	CTLA4	−0.037	2.15 × 10–1	0.02018994	0.52490196
	LAG3	−0.14	3.09 × 10–6^***^	−0.11585047	0.00025198^**^
	TIM-3(HAVCR2)	0.133	9.24 × 10–6^***^	0.18444976	4.6761E-09^***^
	GZMB	−0.084	5.49 × 10–3^*^	−0.03822992	0.22850157

Cor, R value of Spearman’s correlation; None, correlation without adjustment. Purity, correlation adjusted by purity

Abbreviations: *TAM* tumour-correlated macrophage, *Tfh* follicular helper T cell, *Th* T helper cell, *Treg* regulatory T cell

^*^P < .01

^**^P < .001

^***^P < .0001

In addition, the protein expression level of eIF4E can be evaluated in clinical samples from the HPA database. The IHC images showed that eIF4E showed moderate staining in BRCA (Fig. 7B). At the same time, we verified that the expression levels of eIF4E were significantly related to immune cell markers in the same BRCA patients, including those for TAMs (IL10), M2 macrophages (CD163), Th1 cells (STAT1), Th2 cells (GATA3, STAT6), Th17 cells (STAT3) and Tregs (STAT5B). Among these, GATA3, STAT3 and STAT5B were moderately stained and the others were weakly positive. The difference in the expression of immune markers in the tumor tissues of patients with BRCA was further investigated.

**Fig. 8:**
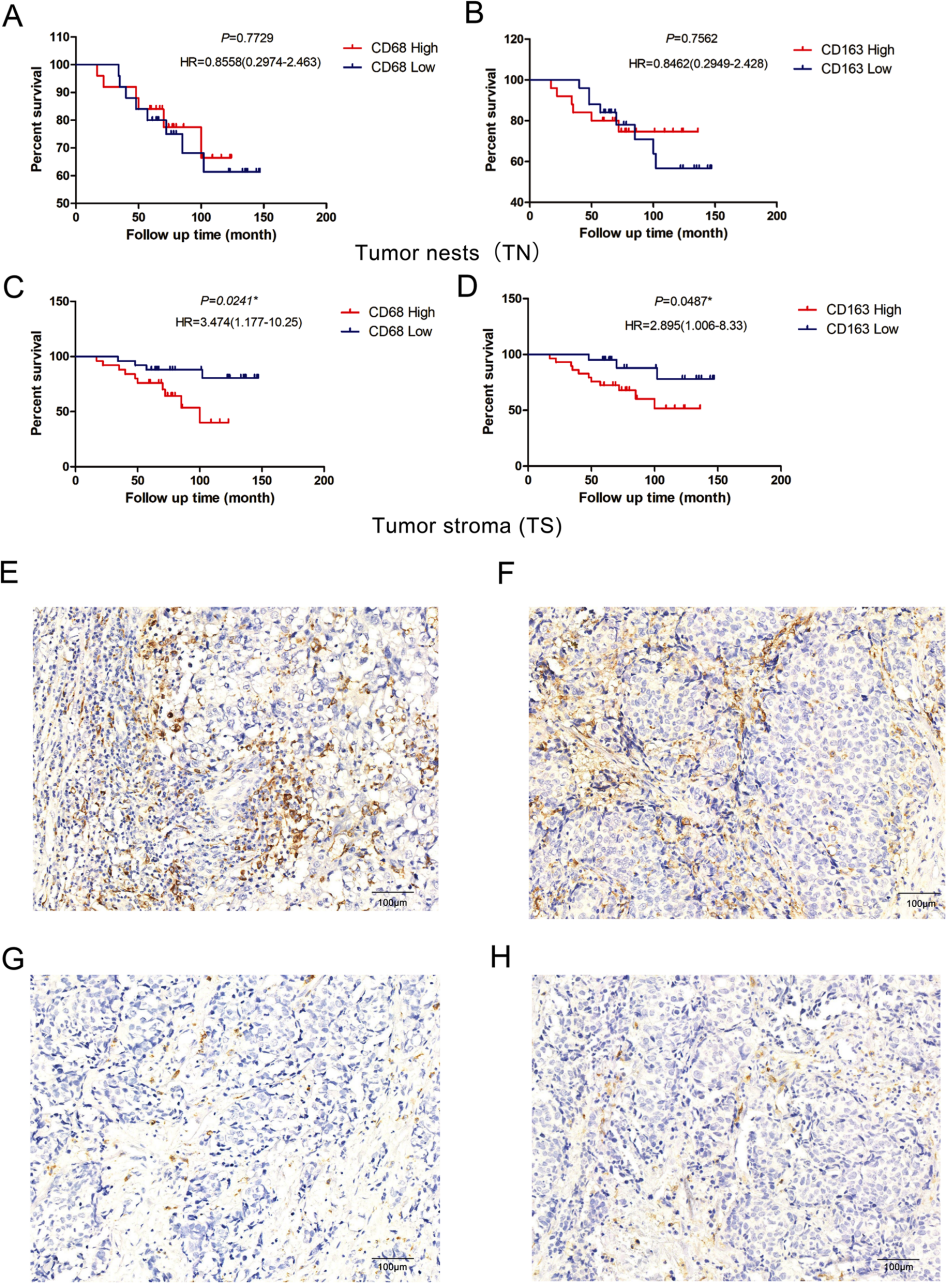
OS curves based on macrophage properties and the density and distribution pattern of macrophage infiltration characterized by CD68^+^ and CD163^+^ immunoreactivity in the tumor nest (TN) and tumor stroma (TS). **A–D** Overall survival curves by CD68^+^ positive macrophage infiltration into the tumor nest (**A**) and stroma (**C**). Overall survival curves by CD163^+^ positive M2 macrophage infiltration into the tumor nest (**B**) and stroma (**D**). Representative images of high density of CD68^+^ staining (**E**) and CD163^+^ staining (**F**) in TN and TS. Representative images of low density of CD68^+^ staining (**G**) and CD163 staining (**H**) in TN and TS. Scale bar, 100 μm

To further verify the results of the database analysis, we stained select BRCA samples by IHC for the TAM macrophage marker (CD68) and M2 macrophage marker (CD163) and performed clinicopathological analysis (Fig. 8). The results showed that the infiltration densities of CD68^+^ and CD163^+^ in BRCA nests were 15.2 ± 8.23 and 21.75 ± 9.18 per field, respectively; the infiltration densities of CD68^+^ and CD163^+^ in BRCA stroma were 20.59 ± 11.07 and 30.87 ± 12.95 per field, respectively. Pearson correlation tests showed that the infiltration densities of CD68^+^ and CD163^+^ macrophages in the TS was negatively correlated with survival time (*r* = − 0.34, *P* = 0.016; *r* = − 0.283, *P* = 0.047), but there was no significant difference in the TN. Kaplan–Meier survival curve analysis showed that the OS of patients with high densities of CD68^+^ and CD163^+^ in TS was significantly shorter than that of patients with low densities (*P* < 0.05) (Fig. 8A-D). The analysis of CD68^+^ and CD163^+^ macrophage infiltration densities and clinicopathological parameters showed that the number of patients with a high infiltration density of the M2 macrophage marker CD163^+^ in the TS was significantly higher than that of patients with a low infiltration density in TNM stage III + IV (*P* < 0.05), but there was no significant difference in age, tumor diameter, lymph node metastasis or grade, as shown in Table 2.

**Table 2:** The differences of postoperative clinical data between high and low expression of CD68^+^ and CD163^+^

Variables	CD68^**+**^				CD163^**+**^			
	Low	High	χ^**2**^	***P***	Low	High	χ^**2**^	***P***
Age			0.76	0.38			0.36	0.54
< 50	17	14			12	19		
≥ 50	8	11			9	10		
Tumor size			0.34	0.56			2.12	0.15
D<3	10	8			10	8		
D ≥ 3	15	17			11	21		
Lymphatic metastasis			0.86	0.35			0.04	0.85
No	16	19			15	20		
Yes	9	6			6	9		
Grade			2.60	0.11			0.09	0.76
I/II	21	16			16	21		
III	4	9			5	8		
TNM stage			1.44	0.23			4.02	0.045*
I + II	24	21			21	24		
III + IV	1	4			0	5		

^*^Statistically significant *p* < 0.05

### Analysis of genes coexpressed with eIF4E in BRCA

To determine the biological significance of eIF4E in BRCA, the functional module of LinkedOmics was used to evaluate the coexpression patterns of eIF4E in the BRCA cohort. As shown in Fig. S2A, 5315 genes (dark red dots) were significantly positively correlated with eIF4E, while 8395 genes (dark green dots) were negatively correlated. The heat map showed the first 50 important genes positively and negatively correlated with PRPF3 (Fig. S2B and C), of which UBE2D3 ubiquitin binding enzyme had the highest positive correlation (*r* = 0.671112, *P* = 5.68e-144). The coexpressed genes are described in detail in Supplementary Table 2.

GO terminology annotations made through GSEA showed that genes coexpressed with eIF4E were mainly involved in chromosome segregation, RNA localization and DNA replication, while genes associated with extracellular structure organization, human immune response and protein localization to the endoplasmic reticulum were inhibited (Fig. S2D, Supplementary Table 3). KEGG enrichment showed that it was mainly concentrated in ubiquitin-mediated proteolysis, RNA transport, cell cycle and other signaling pathways, while ribosome, glycosaminoglycan biosynthesis, cell adhesion molecules and other signaling pathways were inhibited (Fig. S2E, Supplementary Table 4).

In addition, a coexpression network of protein–protein interactions by Differential Net was constructed based on breast-specific data collected from the eIF4E database (Fig. S3A, Supplementary Table 5). The top three central genes were CUL3, heat shock protein 90αA1 (HSP90AA1) and YWHAZ. CUL3 is the core component of the BCR (BTB-CUL3-RBX1) E3 ubiquitin protein ligase complex. The ubiquitin ligase complex mediates the ubiquitination of the target protein and subsequent proteasome degradation [20]; the ubiquitin ligase complex BCR (KLHL25) participates in translation homeostasis by mediating ubiquitination and degradation of hypo-phosphorylated eIF4EBP1 (4E-BP1) degradation [21]. Extracellular heat shock protein 90α (HSP90AA1) has been widely reported to promote tumor cell migration and tumor metastasis in many tumors. It has been observed that extracellular heat shock protein 90α can promote epithelial-mesenchymal transition (EMT) and the migration of cancer cells in BRCA [22]. YWHAZ binds and stabilizes key proteins involved in signal transduction, cell proliferation and apoptosis [23]. Studies have further shown that YWHAZ is involved in drug resistance in BRCA [24].

Finally, the TF (transcription factor)-miRNA regulatory interaction of genes coexpressed with eIF4E was constructed based on the RegNetwork database (Fig. S3B, Supplementary Table 6). The top three TFs were upstream stimulating factor 1 (USF1), CCCTC binding factor (CTCF) and transcription factor YY1. USF1-related studies have shown that USF1 can transcriptionally upregulate the expression of FAK in lung cancer, thus activates the FAK signaling pathway and promotes cell migration [25]. USF1 is involved in the transcription of many proteins and plays an important role as a regulator in many diseases, including tumors [26]. Studies have shown that CTCF expression is involved in tumorigenesis [27] and can be used as a transcription factor to control gene expression by binding to the transcriptional initiation sites (TSSs) of many genes [28]. Some studies have shown that overexpression and binding of the transcription factor YY1 to the BRCA1 promoter inhibits the proliferation and focus formation of MDA-MB-231 cells and inhibits the growth of MDA-MB-231 tumor in nude mice. In addition, a tissue microarray demonstrated that there was a positive correlation between the expression of YY1 and BRCA1 in human BRCA [29, 30].

### Cross-analysis of genes coexpressed with eIF4E and immune marker genes

**Fig. 9:**
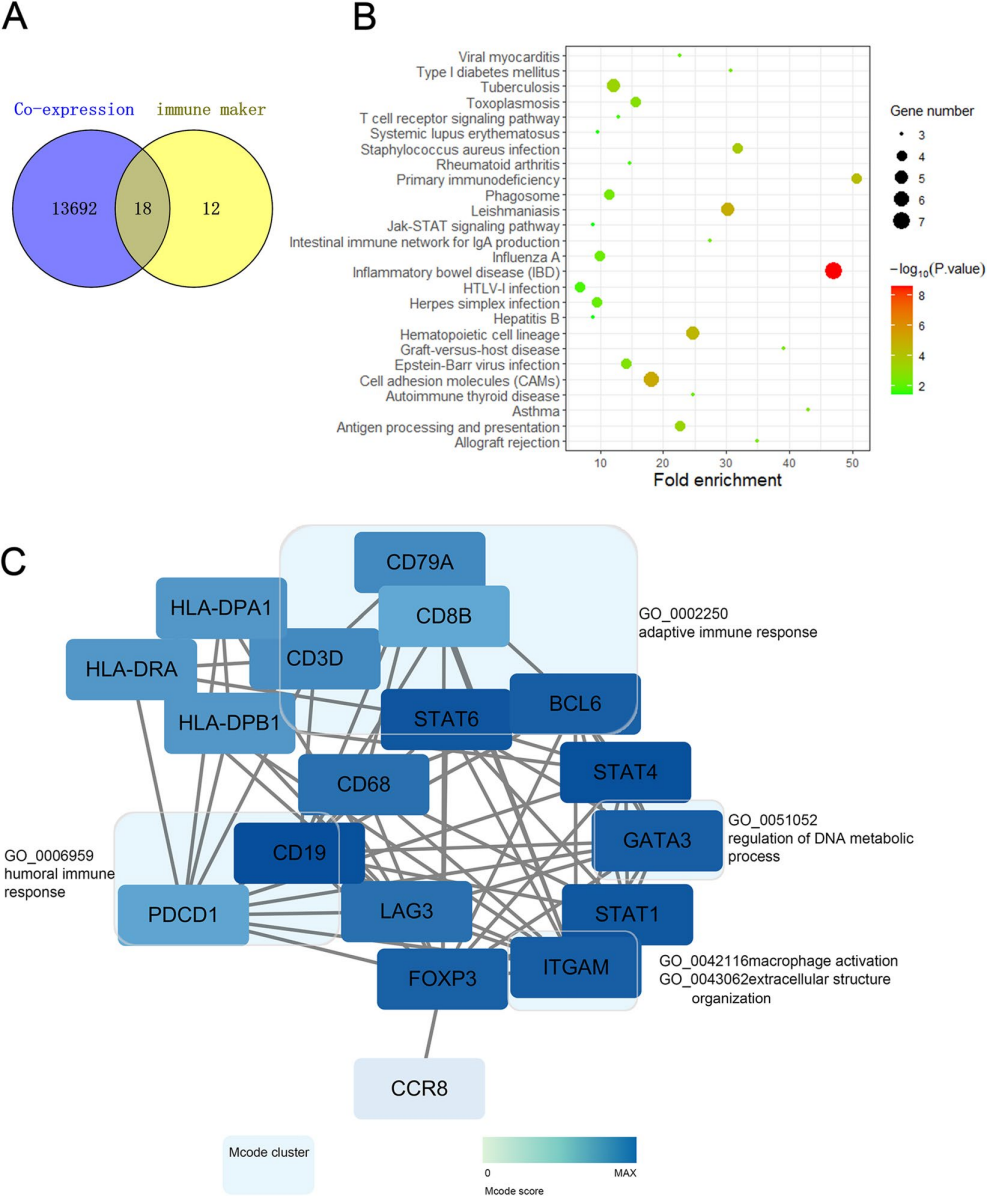
Cross analysis of eIF4E coexpressed genes and immune marker genes. **A** The Venn diagram showing that the eIF4E coexpressed gene overlapped with the immune marker gene. **B** KEGG analysis of genes. **C** Cytoscape and GO analysis of genes

We showed that genes coexpressed with eIF4E were involved in human immune-related biological processes by GO analysis, and KEGG enrichment also showed that they were involved in the cell adhesion molecule pathway, which is related to the expression of cytokines. To further explore the relationship between genes coexpressed with eIF4E and immune infiltration, we performed a cross-analysis of 13,710 coexpressed genes and 30 immune marker genes significantly related to eIF4E. The results showed that there were 18 overlapping genes (Fig. 9A). The interaction of these key genes was analyzed by Cytoscape software and GO analysis. The results showed that the key genes were mainly involved in the human immune response, adaptive immune response, macrophage activation, extracellular structure organization and regulation of DNA metabolic processes (Fig. 9C). KEGG analysis showed that these key genes were mainly involved in inflammatory bowel disease (STAT4, HLA-DPB1, HLA-DRA, STAT6, FOXP3, HLA-DPA1), cell adhesion molecule pathways (CD8B, HLA-DPB1, PDCD1, HLA-DPA1), JAK-STAT signaling pathways (STAT4, STAT6), and the T cell receptor signaling pathway (PDCD1) (Fig. 9B). These results suggested that genes coexpressed with eIF4E were involved in the regulation of tumor immunity and provided strong evidence that eIF4E was an important regulator of immune infiltration in BRCA.

## Discussion

Gene amplification and overexpression of eIF4E are found in many human cancers [31, 32] and correlate with tumorigenesis and progression, as well as with aggressive biological behaviors. To better understand the potential function and regulatory network of eIF4E in BRCA, we conducted bioinformatics analysis of public data. It was validated by in vitro experiments in BRCA cell lines. Samples of invasive BRCA were collected to verify the expression of CD68^+^ and CD163^+^, and pathological data were analyzed. Our results suggested that eIF4E was upregulated in BRCA. Analysis of the tumor pathological stages indicated that eIF4E expression was higher in stage 3 and nodal metastasis N2 status, indicating that eIF4E was related to the prognosis and higher risk in patients with advanced BRCA. In addition, patients with increased eIF4E expression have poor survival and a high cumulative recurrence rate of BRCA. In vitro, it was confirmed that knocking down eIF4E in BRCA significantly inhibited cell migration and invasion. Interestingly, the results of TP53 mutation analysis showed that the expression level of eIF4E was higher in the TP53 non-mutated group than in the mutant group. A recent study demonstrated the heterogeneity of genomic alterations that can occur following mutation of p53. Differences in proliferation, colony formation, and metabolism are associated with aneuploidy but not mutant p53 expression. How the genomic changes that contribute to oncogenic gain-of-function (GOF) phenotypes after WT p53 is lost remains to be further studied [33].

A number of studies found correlations between the presence of infiltrating immune cells in the tumor microenvironment (TME) and the prognosis of many cancers, such as ovarian, renal cell, colorectal, and BRCA [34]. The immune component of the TME consists of predominantly CD4^+^ and CD8^+^ T cells, DCs, macrophages, and Tregs [35]. However, the immune system not only inhibits the growth of cancer cells by destroying cancer cells or inhibiting their outgrowth but also establishes the conditions of the tumor microenvironment to promote tumor growth [34]. In general, T cell infiltration portends a better outcome [36]. Infiltration by mature, active DCs into tumors confers an increase in immune activation and recruitment of disease-fighting immune effector cells and pathways. Neutrophils infiltrating into the primary tumor interact with circulating tumor cells (CTCs) in the bloodstream and are involved in the establishment of a metastatic niche [37, 38]. Circulating monocytes are recruited into breast tumors through chemotactic signals and then differentiate into TAMs to promote tumor growth and metastasis [39, 40]. TAMs are transformed into M2 macrophages to enhance tumor angiogenesis in advanced tumors [41]. In BRCA, TAMs can also secrete matrix metalloproteinase 9 (MMP9) and matrix metalloproteinase 2 (MMP2) to degrade the extracellular matrix. M2 macrophages can produce high levels of MMP, promote extracellular matrix (ECM) degradation, and stimulate tumorigenesis, cancer cell invasion and metastasis by activating EMT [42, 43]. We studied the correlation between eIF4E and immune cell infiltration in BRCA-Luninal and BRCA-Basal by immune infiltration analysis. The results showed that eIF4E was significantly related to macrophage infiltration in BRCA-Basal, and significantly related to the infiltration of B cells, CD8^+^ T cells, macrophages, neutrophils and dendritic cells in BRCA-Luminal. Macrophage infiltration was consistent with the expression of eIF4E in basal and luminal subtypes. Proportional risk model analysis showed that eIF4E acted as an independent prognostic factor in the presence of a variety of infiltrating immune cells in BRCA in general and in luminal subtypes; macrophage infiltration acted as an independent prognostic factor in BRCA-Basal. Traditionally, BRCA has been considered an immune-silent cancer type that is less likely to benefit from immunotherapy. Increasing evidence, however, indicates that different molecular subtypes have different immune infiltration, and TNBC is believed to be a more immunogenic subtype [44, 45]. H. Raza Ali et al. found that higher proportions of certain immune cell types were associated with a greater risk of relapse (or greater chemotherapy response), whereas others were associated with a lower risk and that these associations were often different according to the estrogen receptor (ER) status of the tumor. For example, in ER-negative disease, tumors lacking immune infiltration were associated with the poorest prognosis, whereas in ER-positive disease, their prognosis was between high- and low-infiltration tumor patients [46]. Similarly, in ER-positive disease, both M0 and M2 macrophages were associated with poorer outcome, with a similar pattern in ER-negative disease [47]. Our results indicate that eIF4E is positively correlated with macrophage infiltration in BRCA.

In addition, further correlation analysis between eIF4E and immune markers showed that eIF4E could regulate the tumor infiltrating immune cell pattern in the TME of BRCA. We observed a positive correlation between eIF4E and markers of TAM and M2 macrophages (including CCL2, CD68, IL10, CD163, VSIG4 and MS4A4A) (Table 1), indicating that eIF4E has a role in regulating TAM polarization. To verify the effect of macrophage polarization on BRCA, we analyzed the immunohistochemistry and pathology data of the collected cases. The results of IHC analysis showed that CD68^+^ and CD163^+^ were significantly increased and correlated with poor prognosis in BRCA patients, and there was a significant difference in TNM staging between the CD163^+^ high infiltration density group and the CD163^+^ low infiltration density group in TS (*P* < 0.05), which indicated that TAM polarization and TS infiltration promoted the malignant progression of BRCA. In addition, we found that eIF4E levels in BRCA were correlated with markers of Treg cells and exhausted T cells (FOXP3, CCR8, STAT5B, PD-1, LAG3 and TIM-3), suggesting that eIF4E might inhibit T cell-mediated immunity by promoting the Treg response. In addition, the expression of eIF4E was positively correlated with STAT3 and STAT6. Related studies have shown that some signaling molecules are involved in M2 polarization of macrophages, such as PI3K/AKT-ERK signaling, STAT3, HIF1α, and STAT6. These results suggested that eIF4E might regulate tumor macrophage infiltration in BRCA, which would have effects on the tumor microenvironment.

**Fig. 10:**
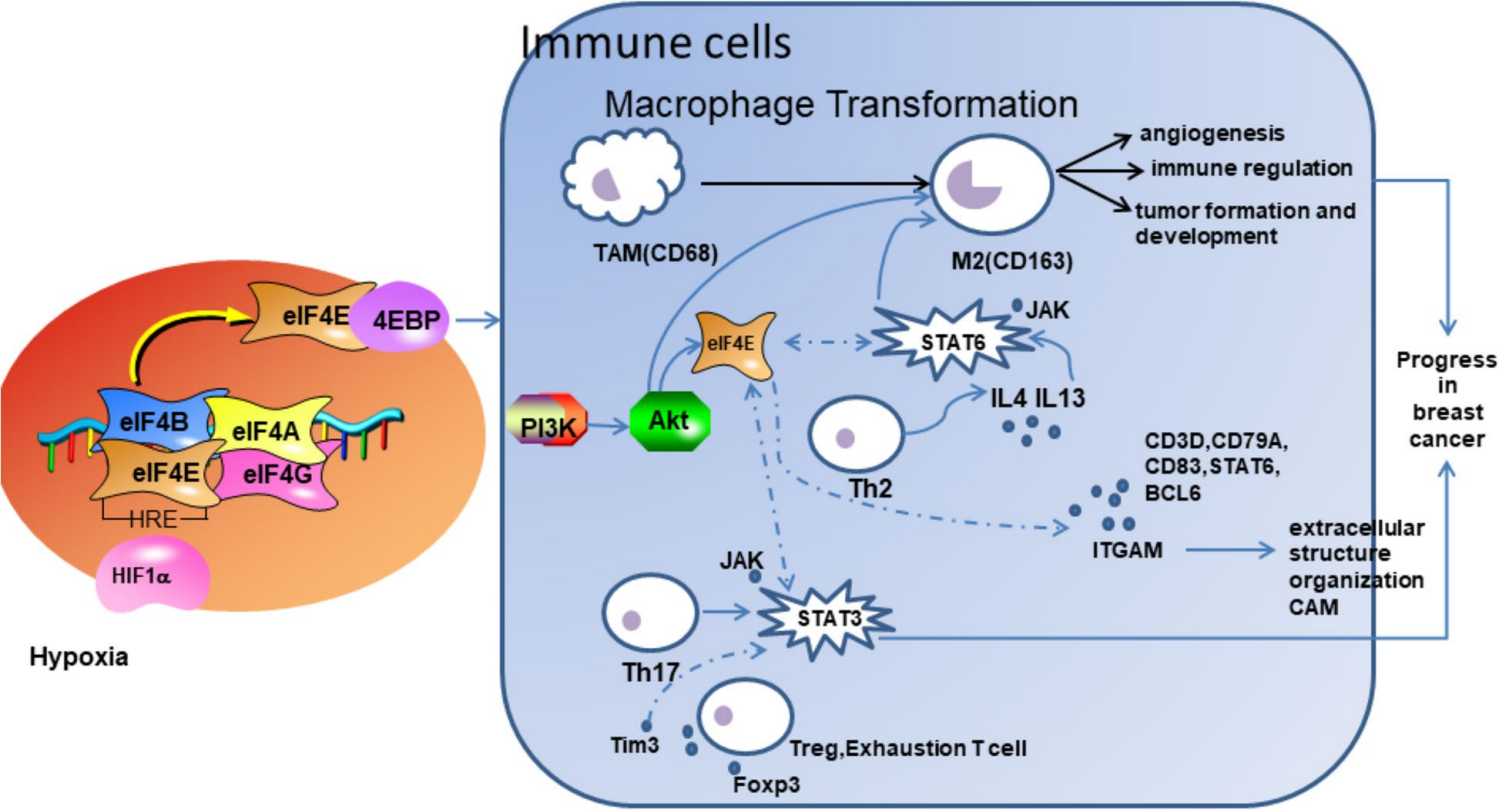
The role of eIF4E in regulating tumor macrophage infiltration in breast cancer

The gene function and pathway enrichment of coexpressed genes and immune marker genes significantly related to eIF4E also showed that they were involved in tumor-related pathways such as the cell adhesion molecule pathway, JAK-STAT signaling pathway, immune-related biological processes such as adaptive immune response, macrophage activation, extracellular structure and regulation of DNA metabolism. Taken together, these results highlighted the potential ability of eIF4E to regulate the recruitment and activation of immune cells in BRCA (Fig. 10).

## Conclusions

In conclusion, eIF4E expression is elevated in BRCA and leads to poor prognosis. eIF4E is a valuable prognostic biomarker. High expression of eIF4E may regulate macrophage infiltration into the tumor and may participate in macrophage M2 polarization.

## Supplementary Information

**Additional file 1: Figure S1.** Correlation between eIF4E and prognosis of various types of cancer Correlation between eIF4E and prognosis of various types of cancer in the GEPIA.

**Additional file 2: Figure S2.** eIF4E co-expression genes in BRCA (LinkedOmics). (A) The global eIF4E highly correlated genes identified by Pearson test in BRCA cohort. (B-C) Heat maps showing top 50 genes positively and negatively correlated with eIF4E in BRCA. Red indicates positively correlated genes and blue indicates negatively correlated genes. (D-E) Significantly enriched GO annotations and KEGG pathways of eIF4E in BRCA cohort. (F)Bar chart of Biological process categories, Cellular component categories and Molecular function categories.

**Additional file 3: FigureS3.** Protein-protein interaction (PPI) and Transcription factor-miRNA (TF-miRNA) regulatory network of eIF4E co-expressed genes (A) The breast-specific PPI network of significantly eIF4E co-expression genes. (B)TF-miRNA coregulatory network of significantly eIF4E co-expression genes.

**Additional file 4: Table S1.** Geo database data set GSE109169 differentially expressed genes.

**Additional file 5: Table S2.** eIF4E co expressed differential genes.

**Additional file 6: Table S3.** eIF4E co-expression gene GO_BP enrichment analysis.

**Additional file 7: Table S4.** Enrichment analysis of eIF4E co-expression gene KEGG.

**Additional file 8: Table S5.** eIF4E co-expression of PPI network in breast.

**Additional file 9: Table S6.** eIF4E co-expression TF-miRNA network.

## Data Availability

The GSE109169 datasets generated during and analyses during the current study are available in the GEO database(http://www.ncbi.nlm.nih.gov/geo/). Oncomine database analysis during the current study are available in https://www.oncomine.org/resource/login.html.PrognoScan database analysis during the current study are available in http://www.abren.net/PrognoScan/. Kaplan-Meier plotter analysis during the current study are available in http://kmplot.com/analysis/. TIMER database analysis during the current study are available in https://cistrome.shinyapps.io/timer/. GEPIA database analysis during the current study are available in http://gepia.cancer-pku.cn/index.html. LinkedOmics database analysis during the current study are available in http://www.linkedomics.org/login.php. Networkanalyst database analysis during the current study are available in https://www.network analyst.ca/. Human protein Altas database analysis during the current study are available in https://www.proteinatlas.org/. In addition,since pathological and clinical data involve patient privacy, if you want to obtain it or other information, please contact the corresponding author.

## Abbreviations

eIF4E: Eukaryotic translation initiation factor 4E
eIF4F: Protein initiation factor complex
OS: Overall survival
TS: Tumor stroma
TN: Tumor nests
TNBC: Triple negative breast cancer
CHOL: Cholangiocarcinoma
COAD: Colon adenocarcinoma
ESCA: Esophageal carcinoma
HNSC: Head and neck squamous cell carcinoma
LIHC: Liver hepatocellular carcinoma
LUAD: Lung adenocarcinoma
LUSC: Lung squamous cell carcinoma
STAD: Stomach adenocarcinoma
UCEC: Uterine corpus endometrial carcinoma
THCA: Thyroid carcinoma
KIRP: Kidney renal papillary cell carcinoma
KIRC: Kidney renal clear cell carcinoma
DSS: Disease Specific Survival
RFS: Relapse-free survival
DCs: Dendritic cells
Tregs: Regulatory T cells
HSP90AA1: Heat shock protein 90αA1
GO: Gene ontology
GSEA: Gene set enrichment analysis
TF: Transcription factor
USF1: Upstream stimulating factor1
CTCF: CCCTC binding factor
TSSs: Transcriptional initiation site
PI3K: Phosphatidylinositol 3-kinase
AKT: Also known as protein kinase B, PKB
mTOR: Mechanical / mammalian target of rapamycin
RAS: Rat sarcoma
MAPK: Mitogen activated protein kinase
MNK: MAPK interacting kinase
HRE: Hypoxia response
TNF: Tumor necrosis factor
FGF: Fibroblast growth factor
MMP9: Matrix metalloproteinase 9
MMP2: Matrix metalloproteinase 2
EMT: Epithelial-mesenchymal transition
ECM: Extracellular matrix
GOF: Oncogenic gain-of-function
IHC: Immunohistochemical
ER: Estrogen receptor
CTCs: Circulating tumor cells
TME: Tumor microenvironment
