# Supplementary figures and images for: High expression of eIF4E is associated with tumor macrophage infiltration and leads to poor prognosis in breast cancer
Source: BMC Cancer. 2021 Dec 7;21:1305. doi: 10.1186/s12885-021-09010-0 (PMC8650334; doi:10.1186/s12885-021-09010-0)

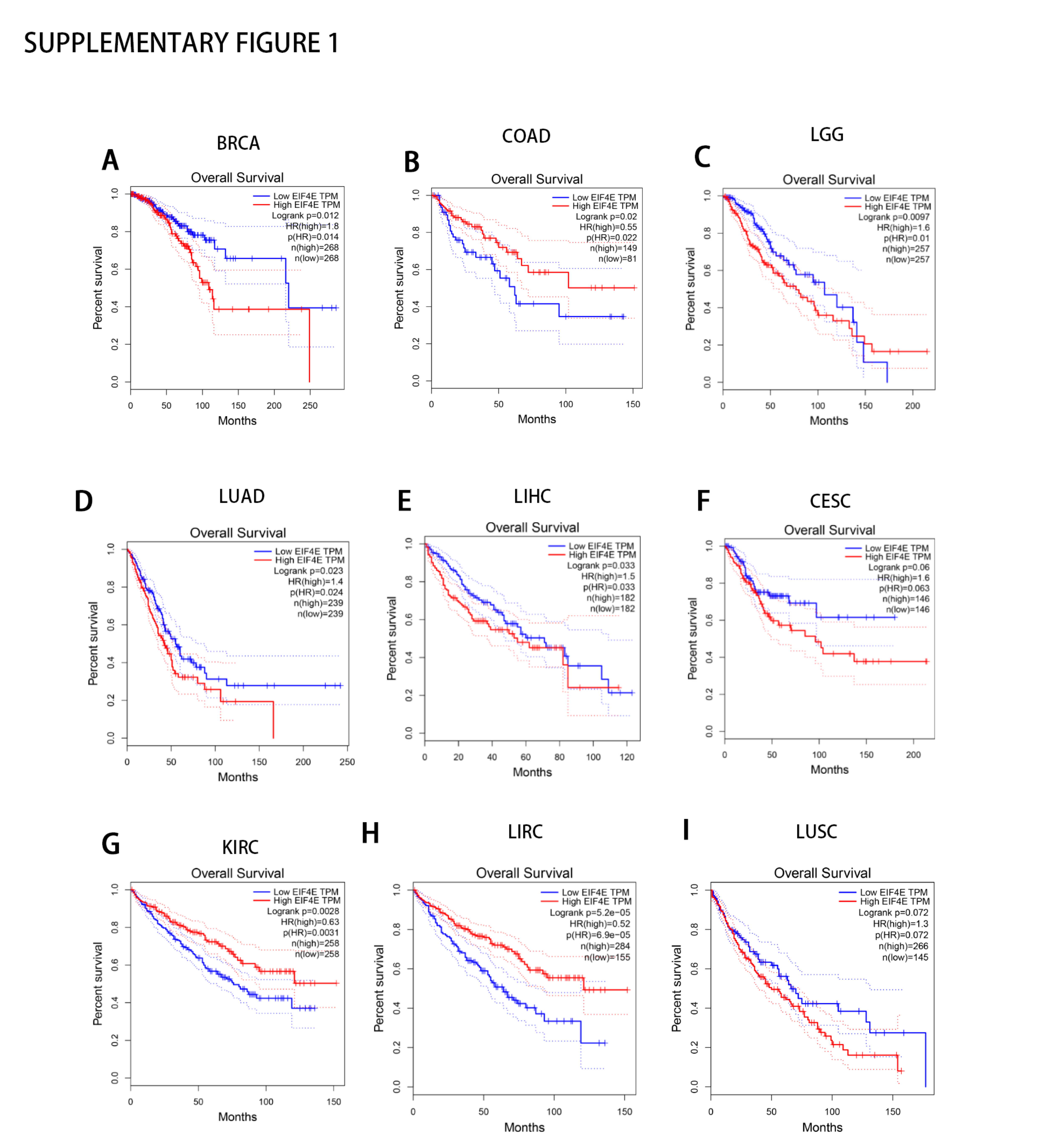

Supplement: Supplementary file 1 — Additional file 1: Figure S1. Correlation between eIF4E and prognosis of various types of cancer Correlation between eIF4E and prognosis of various types of cancer in the GEPIA. [file 12885_2021_9010_MOESM1_ESM.jpg]

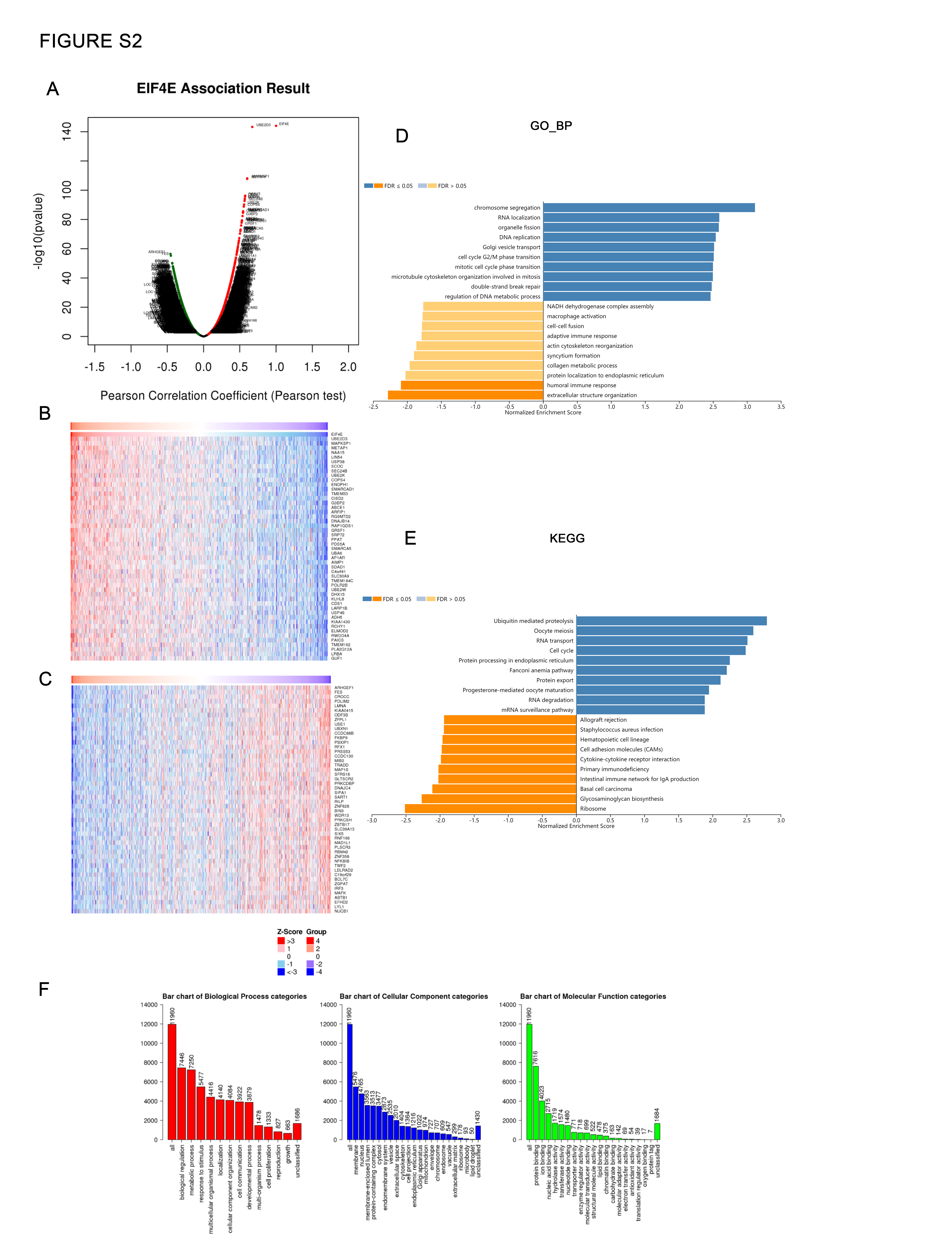

Supplement: Supplementary file 2 — Additional file 2: Figure S2. eIF4E co-expression genes in BRCA (LinkedOmics). (A) The global eIF4E highly correlated genes identified by Pearson test in BRCA cohort. (B-C) Heat maps showing top 50 genes positively and negatively correlated with eIF4E in BRCA. Red indicates positively correlated genes and blue indicates negatively correlated genes. (D-E) Significantly enriched GO annotations and KEGG pathways of eIF4E in BRCA cohort. (F)Bar chart of Biological process categories, Cellular component categories and Molecular function categories. [file 12885_2021_9010_MOESM2_ESM.jpg]

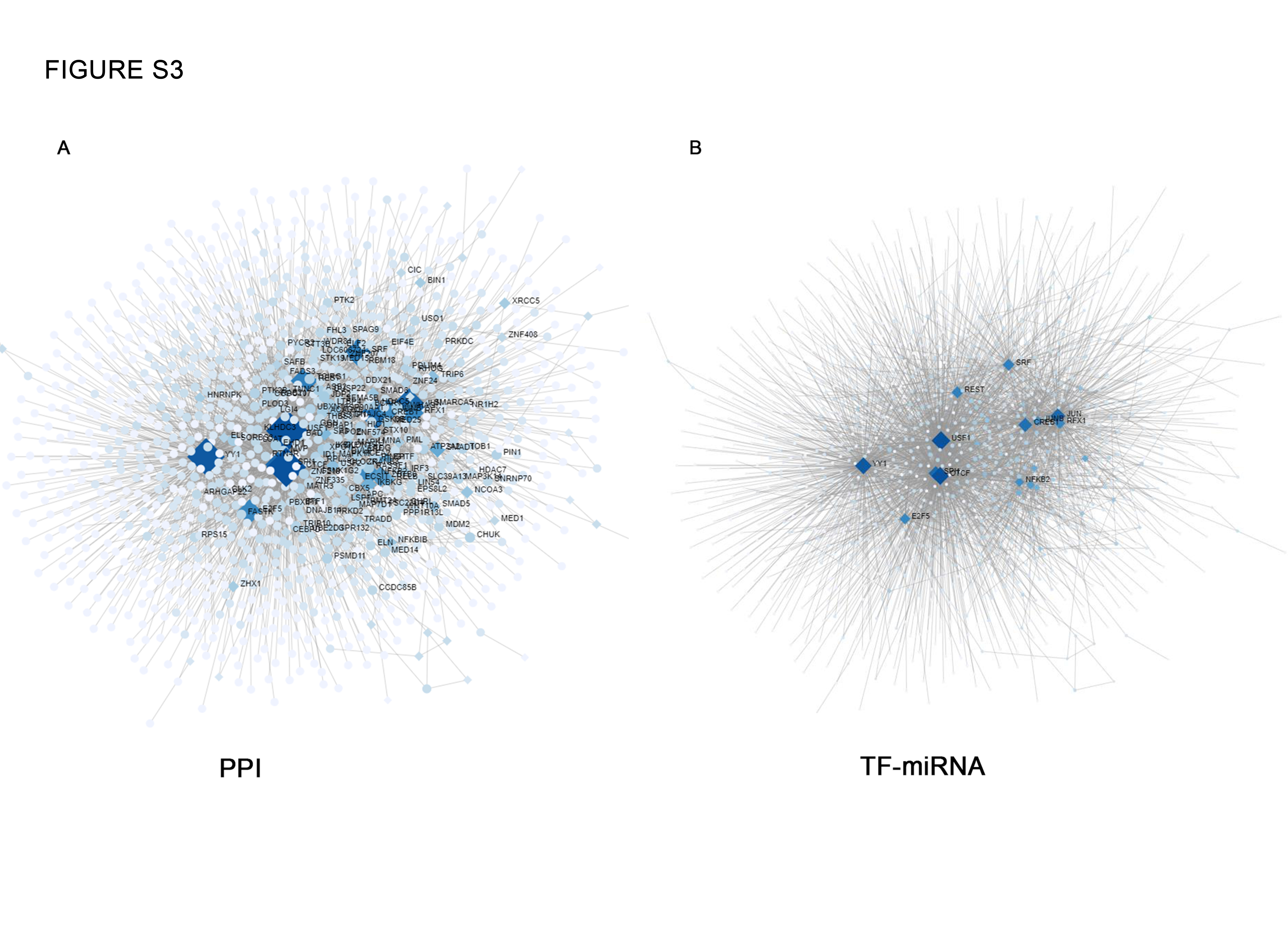

Supplement: Supplementary file 3 — Additional file 3: FigureS3. Protein-protein interaction (PPI) and Transcription factor-miRNA (TF-miRNA) regulatory network of eIF4E co-expressed genes (A) The breast-specific PPI network of significantly eIF4E co-expression genes. (B)TF-miRNA coregulatory network of significantly eIF4E co-expression genes. [file 12885_2021_9010_MOESM3_ESM.jpg]
